# Accelerated Radiotherapy for Complicated Bone Metastases: SHARON Bone Randomized Phase III Trial Shows Non-Inferiority Compared to Standard Palliative Fractionation (NCT03503682)

**DOI:** 10.3390/cancers17122000

**Published:** 2025-06-16

**Authors:** Alice Zamagni, Giambattista Siepe, Dino Gibertoni, Costanza M. Donati, Francesco Cellini, Francesco Fiorica, Donato Pezzulla, Francesco Deodato, Filippo Candoli, Silvia Bisello, Erica Scirocco, Stefania Manfrida, Milena Gabbani, Savino Cilla, Gabriella Macchia, Alessio G. Morganti

**Affiliations:** 1 Radiation Oncology, IRCCS Azienda Ospedaliero-Universitaria di Bologna, 40138 Bologna, Italy; giambattista.siepe@aosp.bo.it (G.S.); costanzamaria.donati@unibo.it (C.M.D.); filippo.candoli@studio.unibo.it (F.C.); alessio.morganti2@unibo.it (A.G.M.); 2 Department of Medical and Surgical Sciences (DIMEC), University of Bologna, 40126 Bologna, Italy; scirocco.erica@gmail.com; 3 Radiation Oncology, Azienda USL-IRCCS di Reggio Emilia, 42123 Reggio Emilia, Italy; 4 Epidemiology and Statistics, IRCCS Azienda Ospedaliero-Universitaria di Bologna, 40138 Bologna, Italy; dino.gibertoni@aosp.bo.it; 5 Radioterapia Oncologica, Dipartimento di Diagnostica per Immagini, Fondazione Policlinico Universitario “Agostino Gemelli” IRCCS, 00168 Roma, Italy; francesco.cellini@policlinicogemelli.it (F.C.); stefania.manfrida@policlinicogemelli.it (S.M.); 6 Dipartimento Universitario Diagnostica per Immagini, Radioterapia Oncologica ed Ematologia, Università Cattolica del Sacro Cuore, 00168 Roma, Italy; 7 Department of Clinical Oncology, AULSS 9 Scaligera, 37045 Verona, Italy; francesco.fiorica@aulss9.veneto.it (F.F.); milena.gabbani@aulss9.veneto.it (M.G.); 8 Radiation Oncology Unit, Responsible Research Hospital, 86100 Campobasso, Italyfrancesco.deodato@unicatt.it (F.D.); gabriella.macchia@responsible.hospital (G.M.); 9 Istituto di Radiologia, Università Cattolica del Sacro Cuore, 00168 Roma, Italy; 10 Radiation Oncology Unit, Azienda Ospedaliero-Universitaria delle Marche, 60126 Ancona, Italy; silvia.bisello@ospedaliriuniti.marche.it; 11 “The Sean M. Healey and AMG Center for ALS”, Massachusetts General Hospital, Harvard Medical School, Boston, MA 02115, USA; 12 Medical Physics Unit, Responsible Research Hospital, 86100 Campobasso, Italy; savino.cilla@responsible.hospital

**Keywords:** bone metastases, palliative radiation therapy, hypofractionation

## Abstract

**Objective:** The SHARON (Short course RadiatiON therapy for palliative treatment) Bone trial is a phase III randomized non-inferiority multicentric study comparing symptom relief for complicated bone metastases (BMs) achieved through hypofractionated accelerated palliative radiotherapy (RT) to a standard RT regimen. **Methods**: Eligible participants were adults with ECOG PS ≤ 3 who were referred for palliative RT for painful BMs. Patients were assigned to receive either 30 Gy delivered in 10 daily fractions or 20 Gy in 4 fractions over two consecutive days. The primary outcome was pain relief one month post-treatment. Pain relief and adverse events were also evaluated at 2, 3, 6, and 12 months after RT. This trial was registered at clinicaltrials.gov (NCT03503682). **Results:** Between February 2018 and November 2021, 83 patients were enrolled (30 Gy: 41; 20 Gy: 42). In the standard RT group, five patients did not complete the prescribed RT, while none in the experimental arm discontinued treatment (*p* = 0.026). Due to early mortality, the primary endpoint was evaluable in 73 patients (35 and 38 in the standard and experimental arms, respectively). The rate of complete pain response at one month was 22.9% and 28.9% in the 30 Gy and 20 Gy arms, respectively (*p*: 0.571). The overall pain response rates, which included complete and partial responses, were 74.3% and 78.9% in the 30 Gy and 20 Gy arms, respectively (*p* = 0.638), when considering at least a 2-point reduction in the numerical rating scale. In both arms, 4.8% of patients experienced Grade >2 toxicity. **Conclusions**: Administering 20 Gy in four fractions twice a day is non-inferior to the standard 30 Gy delivered in 10 fractions for pain relief in the context of complicated BMs. Furthermore, this regimen demonstrated comparable safety in terms of acute toxicity, with a lower incidence of definitive interruptions of radiotherapy.

## Simple Summary

The SHARON Bone phase III randomized trial demonstrated the non-inferiority of a hypofractionated accelerated palliative radiation therapy regimen (20 Gy in 4 fractions over two consecutive days) compared to the standard treatment for symptom palliation of painfully complicated bone metastases. The experimental regimen demonstrated clinically relevant lower radiation treatment discontinuation without increasing acute and late toxicities. Thus, the SHARON regimen should be considered a safe and practical option for fractionated palliative radiation therapy in both hospitalized and outpatient settings, as well as for patients with poor performance status and those concurrently receiving systemic therapies.

## 1. Introduction

Bone metastasis (BM) is a prevalent occurrence in the context of cancer metastasis, alongside the liver and lungs [1], with the potential for metastatic involvement in a wide array of cancer types [2]. BMs profoundly impact the quality-of-life of afflicted individuals, manifesting as debilitating symptoms often characterized by pain and complications related to tissue invasion. These complications may lead to skeletal-related events, including pathological fractures and spinal cord compression.

Radiation therapy (RT) is a pivotal modality for alleviating tumor-related symptoms and represents the standard of care in managing BMs due to its capacity to deliver sustained pain relief with minimal toxicity and reasonable cost-effectiveness. Notably, for uncomplicated BMs, the existing ESTRO guidelines advocate the use of a single 8 Gy fraction as the preferred treatment modality [3]. However, the optimal approach for managing complicated BMs remains poorly defined. Both single-dose RT and fractionated RT schedules are considered viable options in this scenario [4,5].

In the context of palliative RT, short treatment protocols are often favored, given the compromised clinical condition of the patients. This evidence supports the possibility of achieving symptomatic relief with relatively low RT doses, reducing the risk of toxicity, and facilitating the adoption of accelerated hypofractionated treatments.

Since 2012, in the context of the SHARON (SHort course Accelerated RadiatiON therapy for palliative treatment) PROJECT, our investigations have explored the effectiveness of a twice-daily palliative regimen (18–20 Gy in 4 fractions, bid) in a diverse range of clinical settings, including the treatment of bone and brain metastases, as well as primary and secondary tumors in the esophagus, thorax, abdomen, and head-neck regions [6,7].

For complicated BMs, our work encompassed a phase I dose escalation trial, followed by a subsequent phase II trial [8]. These trials demonstrated a notable absence of Grade ≥ 2 acute toxicities, even up to a total dose of 20 Gy. Furthermore, we observed rates of complete symptom remission, partial symptomatic response, and symptom progression of 32.0%, 52.0%, and 8.0%, respectively.

Consequently, we conceived a phase III trial aimed at evaluating the non-inferiority of this regimen when compared to the prevailing standard palliative RT regimen at the time, which consisted of 30 Gy in 10 fractions. The non-inferiority of effectiveness was evaluated as a clinically relevant objective, given the additional advantages of the SHARON schedule in terms of logistics for patients and RT departments and the equivalent tolerability derived from previous phase I and II trials. We hypothesized a 20% relative reduction in the response to pain treatment as a clinically relevant non-inferiority margin. This paper aims to present the findings of our trial, focusing on efficacy and tolerability, which represent the primary and secondary endpoints. A separate, dedicated publication will report the more complex and longitudinal analyses of the quality-of-life outcomes, ensuring sufficient space to appropriately describe the methodology and results.

## 2. Materials and Methods

### 2.1. Study Design

The SHARON Bone trial was a phase 3, open-label, non-inferiority randomized trial conducted across the radiotherapy units of four Italian centers (refer to Supplementary Materials-Figure S1 for the list). The primary objective of this study was to assess the non-inferiority of the SHARON schedule, an accelerated hypofractionated RT regimen (20 Gy in 4 fractions, 5 Gy per fraction, twice a day over two consecutive days), compared to the standard of care (30 Gy in 10 fractions, 3 Gy per fraction, administered in 5 daily fractions per week) for pain palliation in patients with complicated BMs [4].

The secondary objectives of the study were to compare the tolerability of the SHARON schedule with that of the standard treatment and to compare the rates of treatment interruption, re-treatment at the same site, and overall survival (OS) in the two arms.

### 2.2. Participants

Study participants were required to have the following characteristics:

(1) Patients with painful, complicated BMs, defined as those with neuraxis involvement, pathologic fractures, and/or a soft tissue component at the symptomatic site [5].

(2) Pain at the treatment site distinguishable from pain related to other lesions.
(3) A minimum pain intensity of ≥2 when assessed using a numeric rating scale (NRS).
(4) Patients eligible only for palliative treatment and not suitable for definitive treatment.
(5) Eastern Cooperative Oncology Group (ECOG) Performance Status (PS) score less than 4.
(6) Age equal to or greater than 18 years.
(7) No changes made to the analgesic medication during the preceding week.

#### The Exclusion Criteria Were as Follows:

(1) Previous RT performed at the same site.
(2) Pregnancy or lactation.
(3) Patient unavailability for follow-up visits.
(4) The presence of comorbid conditions that, at the discretion of the physicians, made RT inadvisable.

### 2.3. Sample Size Calculation

Based on the available literature, a pain response of 65% is expected among patients undergoing standard treatment [9,10,11]. In designing this trial, the non-inferiority margin was set to a 13% absolute reduction in response among patients in the experimental arm, that is, a 20% relative reduction in response compared to the standard arm. Using the Blackwelder formula [12], 74 patients were required to obtain a power of 65% with a 1-sided alpha = 0.05. The power choice was derived from several considerations based on the results of our previous phase I–II study [8], which demonstrated excellent safety and efficacy in a small cohort of patients, making it relevant to further confirm these results in a larger yet manageable cohort. An attrition rate of 10% was expected; therefore, recruitment was targeted to 82 patients.

### 2.4. Randomization

Ninety-two patients were screened, of whom 83 were enrolled in the trial (see Supplementary Materials-Figure S2 for CONSORT flow diagram). 1:1 treatment allocation was performed by the Radiation Oncology Department at “IRCCS Azienda Ospedaliero-Universitaria di Bologna” using a computer-generated random sequence, and assignment to participants was made by administrative personnel. The participants and researchers were not blinded to the treatment assignments.

### 2.5. Interventions

All patients underwent RT planning CT scans before treatment. The clinical target volume (CTV) was defined as the gross tumor volume (GTV) plus a 2 cm margin in all directions or, in the case of spinal metastases, the entire affected vertebra. The planning target volume (PTV) encompassed the CTV plus a 1-cm margin of upper and lower vertebrae for spinal metastases. Treatment was delivered using 3D-conformal RT, with dose prescription following the ICRU-50 report [13]. More conformal techniques (IMRT, VMAT) were employed for patients when it was considered useful to reduce the risk of toxicity.

Patients were allowed to continue their usual pain medications without restrictions on the type of analgesic used, including NSAIDs and opioids. However, chemotherapy was not permitted concurrently with RT and required a one-week cessation period before and after the treatment. The discontinuation of other systemic treatments, such as immunotherapy or targeted therapies, was planned according to the clinical practice. RT was initiated within 10 days of the first consultation.

### 2.6. Evaluations

During RT, patients had scheduled visits at the first and last fractions and after five fractions for the standard treatment arm. Follow-up visits were planned at 1, 2, 3, 6, and 12 months after RT. Refer to the Supplementary Materials-Table S1 for a summary of the evaluations conducted at each time point. Additionally, telephone follow-up visits were permitted during and after the COVID-19 pandemic.

Pain score (PS) and drug score (DS) (definitions in Supplementary Materials-Table S2) were also recorded [14].

Toxicity was evaluated using the Radiation Therapy Oncology Group (RTOG) and EORTC Common Toxicity Criteria [15].

### 2.7. Primary Endpoint

The primary outcome was pain response at the treatment site, expressed as the difference between pain assessed at baseline and 1 month after treatment. Pain was assessed at each time point using an NRS ranging from 0 (no pain) to 10 (intense pain) and subsequently analyzed using two categories.

The NRS raw data were summarized to obtain a 4-group classification (NRS = 0: complete response; reduction in NRS ≥ 2 points compared to baseline: partial response; change in NRS between −1 and +1 point compared to baseline: stability; increase in NRS ≥ 2 points compared to baseline: progression) and a 2-group classification obtained from the 4-group classification of complete and partial response (overall response) and stability and progression (no response).

Additionally, another pair of 4- and 2-group classifications of response to treatment was obtained from the combination of NRS and DS scores, the criteria for which are described in the Supplementary Materials-Tables S3 and S4.

### 2.8. Secondary Endpoints

The secondary endpoints included the following:

(1) Differences in toxicity between arms.
(2) Treatment interruption and re-treatment at the same site in both the study arms.
(3) Overall survival rates in the two study arms.

### 2.9. Statistical Analysis

For the primary endpoint, both intention-to-treat (ITT) and per-protocol (PP) analyses were carried out to check for the consistency of the results. Patients who did not reach a 1-month follow-up were classified as progressors in the ITT analysis and were excluded from the PP analysis. First, we compared the main clinical characteristics of patients between the two study arms. According to the type and distribution of variables, characteristics were summarized using the mean and standard deviation or frequencies and percentages, and *t*-test, chi-square test, or Fisher’s exact test were used. To evaluate the main objective, pain response was first compared between the two study arms using the four different classifications described above and investigated by a linear mixed model with restricted maximum likelihood (REML) estimation used for the longitudinal analysis of NRS trajectories during the first 3 months of treatment, accounting for study arms, age, ECOG PS, and primary tumor as covariates.

To evaluate differences in toxicity, treatment interruption, and re-treatment at the same site, patients were analyzed using ITT. The proportion of each event was obtained and compared between the arms using Fisher’s exact test. Overall survival was analyzed ITT, with patients observed from treatment initiation until death or the date of the last follow-up. Kaplan-Meier survival curves and log-rank tests were performed to compare OS between the study arms.

All analyses were conducted using Stata v.18.0, with statistical significance set at *p* < 0.05. In multivariable analyses, robust standard errors were obtained according to patient clustering into centers.

## 3. Results

### 3.1. Patients’ Characteristics and Retention in the Study

Between February 2018 and November 2021, 83 patients were enrolled in the SHARON Bone trial. Most patients were enrolled in Bologna (75.9%), while other centers contributed 10.8% (Roma), 8.4% (Legnago), and 4.8% (Campobasso). No statistically significant differences were observed in age, sex, ECOG PS, primary tumor, BM site, or type of complication between the two arms (Table 1).

**Figure 1:**
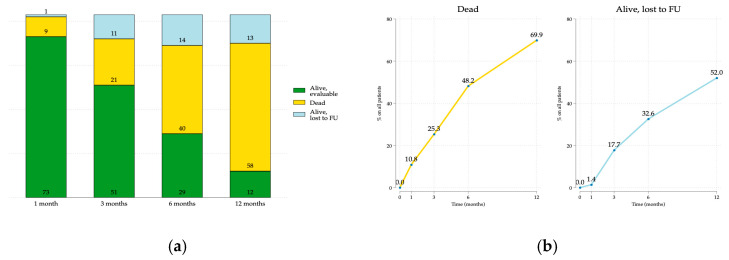
(**a**) patients evaluated at each time point. (**b**) Percentage of patients dead and lost to follow-up (FU) at each time point. The percentage of dead patients is referred to the total number, and the percentage of lost to FU is referred to alive patients at each time point.

At the first follow-up visit (1 month), with figures comparable to expectations, 10.8% of patients died, and 1.2% were lost to follow-up. The two arms remained balanced (n = 38 in the SHARON arm; n = 35 in the standard arm). However, at the following time points, the death rate exceeded expectations, with more than 25% of patients deceased after 3 months and almost 50% after 6 months (Figure 1a,b). The dropout rate was also higher than expected, with 17.7% and 32.6% of surviving patients lost to follow-up at 3 and 6 months, respectively (Figure 1b).

Notably, these data varied significantly when analyzed by period (pre-COVID, COVID, and post-COVID eras; for time interval definitions, see Supplementary Materials-Figure S3), revealing differences in dropout rates over time. In the pre-COVID era, the dropout rates were even lower than expected, peaking at 4.7% at 6 months despite high patient enrollment. In contrast, most dropout cases occurred during and after the COVID-19 pandemic (see Supplementary Materials-Figure S3).

### 3.2. Pain Response

When evaluated in all patients or in the 73 (88.0%) patients with available data at 1 month from treatment, pain response was not significantly different between the study arms (Table 2) using 4-group NRS response (PP: *p* = 0.571, ITT: *p* = 0.501), 2-group NRS (PP: *p* = 0.638, ITT: *p* = 0.436), 4-group NRS + DS response (PP: *p* = 0.549, ITT: *p* = 0.465). and 2-group NRS + DS response (PP: *p* = 0.215, ITT: *p* = 0.155).

Per-protocol, the complete response rates were 22.9% and 28.9% in the standard and experimental arms, respectively. The overall response rates were 74.3% and 78.9% in the standard and experimental arms, aligning closely with the expected figures. Consequently, 25.7% and 21.4% of patients were classified as having “no response ” (i.e., stable disease or progression). A lower proportion of patients with symptomatic progression was found in the experimental arm than in the standard arm (5.2% vs. 14.3%).

As expected, when considering pain medication modifications, the proportions of patients with complete and overall responses decreased, particularly in the standard arm (overall response, 54.3%). The overall response rate in the experimental arm remained high (68.4%). However, no statistically significant difference was found using this classification. Please refer to the Supplementary Materials-Table S5 for the pain response when considering smaller differences in NRS before and after treatment.

**Figure 2:**
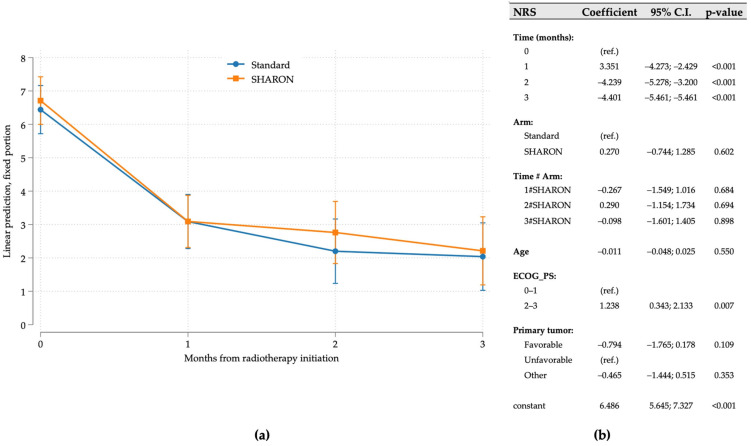
(**a**,**b**) NRS in the first 3 months after treatment estimated using a multivariable linear mixed model (**a**) In the last row of the table (**b**), the constant refers to the model-estimated NRS value for the reference individual, i.e., a patient in the standard arm at time 0, with mean age, ECOG 0–1, and unfavorable primary tumor. Time # Arm: 1#: pre-COVID period, 2#: COVID period; 3#: post-COVID period.

The mixed-effects regression of NRS over the first 3 months, conducted on all 83 patients, displayed no difference between the two study arms. The pain was significantly reduced after 1 month and remained stable thereafter, with overlapping trajectories (Figure 2). Among the covariates included in the model, only ECOG PS was statistically significant, with ECOG values 2–3 estimated at a 1.24 higher NRS than ECOG 0–1.

### 3.3. Toxicity

More than 60% of the patients (61.4%) did not experience any toxicity (Table 3). Toxicity of any grade was observed in 43.9% (18 patients) and 31.0% (13 patients) of patients in the standard and experimental arms, respectively, with no statistically significant difference (*p* = 0.190). For details on the recorded toxicity, refer to the Supplementary Materials-Table S6.

Two Grade 3 toxicities (one hematological and one spinal, respectively, pancytopenia and severe paresthesia) were recorded in the experimental arm, while no Grade 3 toxicity was observed in the standard arm. No Grade 4 toxicity was recorded in the experimental arm compared to two vertebral fractures in the standard arm. The rate of Grade 3 toxicity was 4.8% in the experimental arm, and the rate of Grade 4 toxicity was 4.8% in the standard arm. Notably, the registered Grade 3 and 4 toxicities were unlikely to be radiation-related, as they were temporally related to the start of new chemotherapy lines or evidence of disease progression.

No late-onset toxicity was observed in either arm.

### 3.4. Treatment Interruption

Five patients (6.0%) did not complete RT (Table 3), and all were in the standard arm. The difference in the treatment interruption rate between the standard and experimental arms was statistically significant (*p* = 0.026). Re-irradiation occurred in nine patients (10.8%), six in the standard arm, and three in the experimental arm (*p* = 0.313).

### 3.5. Survival

**Figure 3:**
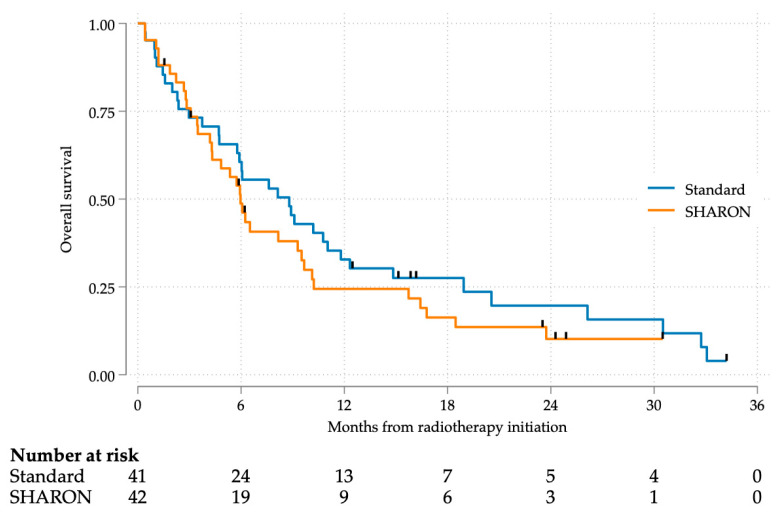
Overall survival.

Mortality rates were very similar in both study arms, with 35 events in each arm, resulting in mortality rates of 85.4% in the standard arm and 83.3% in the SHARON arm. Survival curves revealed that 50% mortality was reached around 6 months after the initiation of RT (Figure 3). The log-rank test for curve comparison was not statistically significant (*p* = 0.335).

## 4. Discussion

The SHARON Bone trial demonstrated the non-inferiority of a hypofractionated accelerated RT schedule, known as the SHARON regimen, compared to standard treatment in palliating symptoms caused by complicated BMs.

This result was maintained even when different pain response classifications were considered and when the pain response was adjusted for analgesic intake.

Another significant finding was the lower rate of treatment interruptions associated with the SHARON schedule, suggesting that this regimen is more feasible for patients. This can be especially valuable for hospitalized patients and those receiving concurrent systemic therapies, where a shorter two-day treatment schedule can be delivered more easily and with less interference with other treatments.

Toxicity was generally low in both arms, and no statistically significant differences were observed. Notably, slightly unexpected Grade 3 and 4 toxicities were observed in the experimental and standard arms. However, an in-depth analysis of these cases revealed that the events were more likely associated with chemotherapy and pre-existing impending fractures than with RT itself.

Although the use of 30 Gy in 10 fractions has declined in recent years in favor of more hypofractionated regimens, it remained a standard comparator for complicated bone metastases at the time the study and its preceding phase I–II trial were designed. Current European guidelines [4] continue to recommend 30 Gy in 10 fractions for specific scenarios, such as pathological fractures or extraosseous tumor extension. We selected this regimen as the control arm because of its high biological effectiveness (EQD2α/β10 = 32.5 Gy; EQD2α/β3 = 36 Gy), ensuring a rigorous non-inferiority test. By demonstrating comparable efficacy to this benchmark, the SHARON regimen would reasonably be expected to perform similarly or better than other commonly used hypofractionated regimens, such as 20 Gy in five fractions (EQD2α/β10 = 23.3 Gy; EQD2α/β3 = 28 Gy).

The pragmatic approach of this study, with minimal exclusion criteria, resulted in a sample that was representative of the general palliative population. The primary endpoint was defined solely based on NRS modification at the treated site, as excluding patients with other painful metastases or considering drug intake modifications might have introduced confounding variables. The relative heterogeneity of patients, including those with poor performance status (ECOG PS 3) and those receiving concomitant systemic therapies, further adds to the relevance of the study findings, as these inclusive criteria mirror real-world clinical scenarios.

Nonetheless, this study has some limitations. In fact, the dropout and mortality rates were underestimated in the sample size calculation, limiting the interpretation of the results beyond the primary endpoint. Importantly, the dropout rate increased during and after the COVID-19 pandemic, reflecting disruptions in healthcare access.

The choice of the standard arm using a 10-day schedule may not fully capture the range of fractionations used in palliative settings [16].

The possibility of using regimens shorter than 10 fractions, even for complicated metastases, is now recognized in international guidelines. For example, the recent ASTRO guidelines [16] propose options such as 8 Gy in a single fraction, 16 Gy in two fractions, or 20 Gy in five fractions, even for patients with spinal cord or cauda equina compression. In this context, our study contributes by proposing an additional alternative for radiation oncologists, which offers the potential effectiveness of a shorter duration of treatment (2 days) and could also be convenient for patients.

Notably, recent guidelines [16] also recommend dose escalation for complex bone metastases in some settings (i.e., oligometastatic disease). While our study predates these guidelines, it remains relevant as it highlights an effective multifractionated regimen for purely palliative purposes. The ASTRO guidelines emphasize the importance of considering factors such as life expectancy, tumor radiosensitivity, and the extent of metastatic disease when deciding on a radiotherapy regimen. In this context, our study provides an alternative for cases in which dose escalation may not be feasible or necessary.

A limitation of our study is that the dropout and mortality rates were underestimated in the sample size calculation, limiting the interpretation of the results beyond the primary endpoint. Importantly, the dropout rate increased during and after the COVID-19 pandemic, reflecting disruptions in healthcare access. Importantly, dropout rates increased during and after the COVID-19 pandemic. This trend reflects a broader phenomenon observed across many oncology trials during that time, attributed to travel restrictions, fear of infection, reduced hospital access, reallocation of clinical resources, and limitations of remote follow-up procedures [17,18].

These challenges likely contributed to the increased number of missed follow-up visits and loss to follow-up in the study population. COVID-19 vaccination status was not collected, as it was not considered relevant to the endpoints of pain response and radiotherapy tolerability in a palliative population with limited life expectancy and short follow-up periods. Infection control measures, including mask-wearing and social distancing, were implemented at all centers during the COVID-19 period according to institutional policies. However, as these procedures were standardized across all sites and were not expected to influence the study endpoints, they were not included as study variables.

Another limitation of this trial is the decision to set the statistical power at 65%, which was made after examining the evidence obtained from our previous phase I–II study [8]. An early study demonstrated excellent safety and efficacy in a cohort of 25 patients, with an overall response rate of 84% and a complete pain response of 32%. Thus, we deemed it necessary to further assess the safety of the 20 Gy regimen in a larger yet manageable cohort to minimize patient exposure and resource demands. However, we acknowledge that the reduced power limits the interpretation of the secondary endpoints and the detection of subtle differences between treatments. Therefore, the results should be interpreted cautiously, especially when used to inform clinical decision-making beyond the primary outcome. Nevertheless, we assume that small differences in efficacy that may not be detectable with our trial sample size are unlikely to have major clinical relevance in the broader context of patient care, especially considering that the experimental regimen offers significant logistical and convenience advantages for patients, such as shorter treatment duration and fewer interruptions, which are particularly valuable in palliative settings.

Thus, while the substandard power limits the ability to detect smaller differences in outcomes, our findings remain valuable and provide a foundation for future larger-scale studies.

Furthermore, it is noteworthy that the method of pain response assessment in this study differed from the approach recommended by current international guidelines [19]. The reason for this variation can be traced back to the fact that this phase III study represents the logical continuation of phase I and II trials, whose protocols were defined before 2010. At that time, the current guidelines had not yet been published.

The use of a two-point reduction in the NRS as a response criterion may have reduced our ability to detect pain reduction, especially in patients with an initial NRS score of two. While we attempted to account for analgesic intake through a complementary DS, we acknowledge that only analgesic class was collected and not the specific drug or dosage, precluding the conversion into oral morphine equivalents as recommended by current guidelines.

However, it is important to emphasize that the main result of this study, namely the non-inferiority of the SHARON regimen, is unlikely to be significantly influenced by the choice of evaluation scales. This is further supported by the fact that different assessment systems were employed, and regardless of these variations, the results were similar. Furthermore, both intention-to-treat and per-protocol analyses were conducted for the primary endpoint, and their consistency supports the robustness of our findings despite the incomplete follow-up data. The alignment between these approaches indicates that the missing data did not substantially bias the treatment effect estimates.

Moreover, to allow a more robust evaluation of the results of the SHARON regimen and specifically to enable sub-analyses in specific subgroups of patients, a prospective observational study in which all patients with complicated bone metastases are enrolled is currently underway at our center. Furthermore, this study included an outcome assessment based on the latest International Bone Metastases Consensus Guidelines, including a pre- and post-treatment analysis of oral morphine equivalents.

Finally, although this paper focused on the overall comparison of the two treatment arms, exploratory subgroup analyses (e.g., by ECOG status, metastasis location, or primary tumor type) are ongoing to better identify which patients might benefit most from the SHARON regimen. These analyses will be reported in future publications.

## 5. Conclusions

In summary, the SHARON regimen is a safe and practical palliative RT regimen. It is suitable for both hospitalized and outpatient settings, particularly when RT departments can accommodate a 6/8-h interval between the two daily fractions. This regimen is also well suited for patients with poor performance status and those concurrently receiving systemic therapies. Additionally, it was associated with significantly fewer treatment interruptions (*p* = 0.026).

It is worth noting that while 8 Gy in a single fraction is generally used for uncomplicated bone metastases, complicated cases, such as those treated in our study, are more commonly managed with fractionated regimens like 20 Gy in five fractions or 30 Gy in ten fractions. In contrast, the SHARON regimen reduces the number of treatment days and hospital visits, suggesting potential cost and resource savings. A formal cost-effectiveness analysis comparing these fractionated options would be valuable in future studies.

Ongoing and future analyses of quality-of-life modifications following the SHARON regimen will provide further insights. Future studies should explore patient stratification based on ECOG PS, life expectancy, primary tumor type, analgesic intake, and baseline NRS to determine the patients most suitable for the SHARON regimen.

Other studies could also investigate the SHARON regimen in comparison to shorter options, such as 8 Gy single fractions, and evaluate its superiority and cost-effectiveness, considering that, at least for patients with spinal cord compression, the results of some studies are already available, showing that a single 8 Gy fraction is non-inferior to multiple fraction regimens in terms of pain relief, neurological outcomes, and survival [20,21,22,23,24].

## Figures and Tables

**Table 1:** Patient characteristics at the baseline visit.

		Treatment Arm		
	All	Standard	SHARON	*p*-Value
	83 (100.0%)	41 (49.4%)	42 (50.6%)	
**Age at randomization**	64.0 ± 11.3	62.7 ± 10.7	65.2 ± 11.8	0.323 *
**Age class**				
<65 y	44 (53.0%)	25 (61.0%)	19 (45.2%)	0.151 ^§^
≥65	39 (47.0%)	16 (39.0%)	23 (54.8%)	
**Sex**				
Male	52 (62.7%)	24 (58.5%)	28 (66.7%)	0.444 ^§^
Female	31 (37.3%)	17 (41.5%)	14 (33.3%)	
**ECOG_PS**				
0–1	57 (68.7%)	30 (73.2%)	27 (64.3%)	0.383 ^§^
2–3	26 (31.3%)	11 (26.8%)	15 (35.7%)	
**Primary tumor**				
Favorable	21 (25.3%)	10 (24.4%)	11 (26.2%)	0.559 ^§^
Unfavorable	40 (48.2%)	22 (53.7%)	18 (42.9%)	
Other	22 (26.5%)	9 (22.0%)	13 (31.0%)	
**Metastases site**				
Spine	38 (45.8%)	20 (48.8%)	18 (42.9%)	0.624 °
Pelvis	23 (27.7%)	9 (22.0%)	14 (33.3%)	
Thorax	17 (20.5%)	10 (24.4%)	7 (16.7%)	
Extremities	5 (6.0%)	2 (4.9%)	3 (7.1%)	
**Type of complication**				
Spinal compression	8 (9.9%)	4 (9.8%)	4 (10.0%)	0.970 °
Nerve compression	18 (22.2%)	10 (24.4%)	8 (20.0%)	
Pathological fracture	11 (13.6%)	5 (12.2%)	6 (15.0%)	
Extraosseous extention	44 (54.3%)	22 (53.7%)	22 (55.0%)	

* *t*-test; ^§^ chi-square test; ° Fisher’s exact test.

**Table 2:** Pain response at 1 month.

Intention-to-Treat Analysis	All 83 (100.0%)	Standard 41 (49.4%)	SHARON 42 (50.6%)	*p*-Value
**NRS response (4 groups)**				
Complete response	19 (22.9%)	8 (19.5%)	11 (26.2%)	0.501
Partial response	37 (44.6%)	18 (43.9%)	19 (45.2%)	
Stable disease	10 (12.0%)	4 (9.8%)	6 (14.3%)	
Progression	17 (20.5%)	11 (26.8%)	6 (14.3%)	
**NRS response (2 groups)**				
Overall response (complete + partial)	56 (67.5%)	26 (63.4%)	30 (71.4%)	0.436
No response (stable + progression)	27 (32.5%)	15 (36.6%)	12 (28.6%)	
**NRS + DS response (4 groups)**				
Complete response	17 (20.5%)	6 (14.6%)	11 (26.2%)	0.465
Partial response	28 (33.7%)	13 (31.7%)	15 (35.7%)	
Stable disease	17 (20.5%)	10 (24.4%)	7 (16.7%)	
Progression	21 (25.3%)	12 (29.3%)	9 (21.4%)	
**NRS + DS response (2 groups)**				
Overall response (complete + partial)	45 (54.2%)	19 (46.3%)	26 (61.9%)	0.155
No response (stable + progression)	38 (45.8%)	22 (53.7%)	16 (38.1%)	
PER-PROTOCOL ANALYSIS	All 73 (100.0%)	Standard 35 (47.9%)	SHARON 38 (52.1%)	*p*-value
**NRS response (4 groups)**				
Complete response	19 (26.0%)	8 (22.9%)	11 (28.9%)	0.571 *
Partial response	37 (50.7%)	18 (51.4%)	19 (50.0%)	
Stable disease	10 (13.7%)	4 (11.4%)	6 (15.8%)	
Progression	7 (9.6%)	5 (14.3%)	2 (5.3%)	
**NRS response (2 groups)**				
Overall response (complete + partial)	56 (76.7%)	26 (74.3%)	30 (78.9%)	0.638
No response (stable + progression)	17 (23.3%)	9 (25.7%)	8 (21.1%)	
**NRS + DS response (4 groups)**				
Complete response	17 (23.3%)	6 (17.1%)	11 (28.9%)	0.549
Partial response	28 (38.4%)	13 (37.1%)	15 (39.5%)	
Stable disease	17 (23.3%)	10 (28.6%)	7 (18.4%)	
Progression	11 (15.1%)	6 (17.1%)	5 (13.2%)	
**NRS + DS response (2 groups)**				
Overall response (complete + partial)	45 (61.6%)	19 (54.3%)	26 (68.4%)	0.215
No response (stable + progression)	28 (38.4%)	16 (45.7%)	12 (31.6%)	

*p*-values from chi-square test, except for * (Fisher’s exact test).

**Table 3:** Secondary outcomes.

	Total Population, n: 83	Standard Arm, n: 41	Sharon Arm, n: 42	*p*-Value °
**Acute toxicity**				0.190
NO	51 (61.4%)	22 (53.7%)	29 (69.0%)	
YES	31 (37.3%)	18 (43.9%)	13 (31.0%)	
N.A.	1 (1.2%)	1 (2.4%)	0 (0.0%)	
**Treatment completed**				0.026
NO	5 (6.0%)	5 (12.2%)	0 (0.0%)	
YES	78 (94.0%)	36 (87.8%)	42 (100.0%)	
**Re-treatment (same site)**				0.313
NO	74 (89.2%)	35 (85.4%)	39 (92.9%)	
YES	9 (10.8%)	6 (14.6%)	3 (7.1%)	

°: Fisher’s exact test.

## Data Availability

The original data presented in this study are openly available in the Zenodo Repository at 10.5281/zenodo.15592109.

## Supplementary Materials

The following supporting information can be downloaded at: https://www.mdpi.com/article/10.3390/cancers17122000/s1; Figure S1: List of participating centers; Figure S2: Consort flow diagram; Table S1: Time points and evaluations; Table S2. Pain and drug score definitions are provided in Table S3. Pain response classification adjusted for drug intake 1 month after treatment initiation is shown in Table S4. Different classifications for each patient when changing the definition of pain response (Table S5). Pain response considering NRS modifications of 1 point (Figure S3). Patients lost to follow-up before, during, and after the COVID pandemic; Table S6. Type and grade of toxicity.
