# Supplementary material for: Accelerated Radiotherapy for Complicated Bone Metastases: SHARON Bone Randomized Phase III Trial Shows Non-Inferiority Compared to Standard Palliative Fractionation (NCT03503682)
Source: Cancers (Basel). 2025 Jun 16;17(12):2000. doi: 10.3390/cancers17122000 (PMC12190215; doi:10.3390/cancers17122000)
Supplement: Supplementary file 1 [file cancers-17-02000-s001.zip › cancers-3613816-supplementary.pdf]

Figure S1. List of participating centres

- IRCCS Azienda Ospedaliero-Universitaria di **Bologna (Promotor Centre)**
- Policlinico A. Gemelli, Università Cattolica del Sacro Cuore, **Roma**
- Fondazione di Ricerca e Cura “Giovanni Paolo II”, U.O. di Radioterapia, **Campobasso**
- AULSS 9 Scaligera, U.O. Radioterapia e Medicina Nucleare, **Legnago**

Figure S2. Consort flow diagram

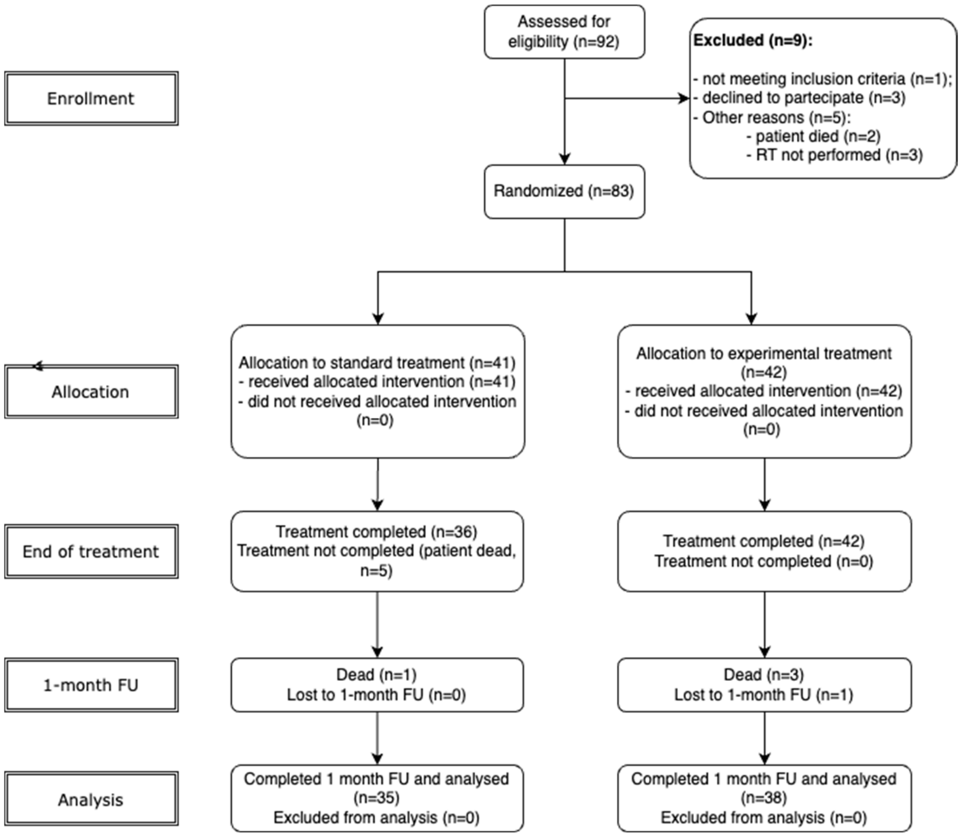

Table S1. Time points and evaluations

	CRF (tumor and patient characteristics)	Questionnaire QLQ-C15-PAL	Questionnaire QLQ-BM22	Pain evaluation (NRS)	Pain score	Drug score	Acute/Late toxicities
Baseline*	X	X	X	X	X	X	
First RT fr				X	X	X	
Last RT fr				X	X	X	X
1-mo FU		X	X	X	X	X	X
2-mo FU		X	X	X	X	X	X
3-mo FU		X	X	X	X	X	X
6-mo FU		X	X	X	X	X	X
12-mo FU		X	X	X	X	X	X

*at randomization
Abbreviations: CRF: case report form; fr: fraction; FU: follow-up; mo: months; NRS: numeric rate score; RT: radiotherapy

Table S2. Pain and drug score definitions (Salazar et al. 2001)

**Pain intensity**		**Drug intensity**	
0	No pain	0	No therapy
1	Mild	1	Non-opioid drugs
2	Moderate	2	Minor opioid drugs
3	Severe	3	Major opioids drugs
**Pain frequency**		**Drug frequency**	
0	No pain	0	No therapy
1	Occasional	1	Less than one time per day
2	Intermittent	2	One time per day
3	Constant	3	≥2 times per day
**Pain score= pain intensity x pain frequency**		**Drug score= drug intensity x drug frequency**	

Table S3. Pain response classification adjusted for drug intake at 1 month from treatment initiation

Criteria for classification	Complete response	Partial response	Stable response	Symptoms progression
**NRS**	$NRS_{1mo}=0$	$NRS_{1mo} \leq NRS_{baseline}-2$	$NRS_{baseline}-1 \leq NRS_{1mo} \leq NRS_{baseline}+1$	$NRS_{1mo} \geq NRS_{baseline}+2$
**NRS+Drug Score (DS)**	$NRS_{1mo}=0$ AND $DS_{1mo} \leq DS_{baseline}$	$NRS_{1mo} \leq NRS_{baseline}-2$ AND $DS_{1mo} \leq DS_{baseline}$<br>OR<br>$NRS_{baseline}-1 \leq NRS_{1mo} \leq NRS_{baseline}+1$ AND $DS_{1mo} < DS_{baseline}$	$NRS_{baseline}-1 \leq NRS_{1mo} \leq NRS_{baseline}+1$ AND $DS_{1mo}=DS_{baseline}$<br>OR<br>$NRS_{1mo}=0$ AND $DS_{1mo} > DS_{baseline}$<br>OR<br>$NRS_{1mo} \leq NRS_{baseline}-2$ AND $DS_{1mo} > DS_{baseline}$	$NRS_{1mo} \geq NRS_{baseline}+2$ AND $DS_{1mo} \geq DS_{baseline}$<br>OR<br>$NRS_{baseline}-1 \leq NRS_{1mo} \leq NRS_{baseline}+1$ AND $DS_{1mo} > DS_{baseline}$

Table S4. Different classifications for each patient when changing the definition of pain response

id	arm	NRS	NRS+DS	Change in classification
16	SHARON	COMPLETE	COMPLETE	
25	SHARON	COMPLETE	COMPLETE	
38	SHARON	COMPLETE	COMPLETE	
42	SHARON	COMPLETE	COMPLETE	
52	SHARON	COMPLETE	COMPLETE	
54	SHARON	COMPLETE	COMPLETE	
55	SHARON	COMPLETE	COMPLETE	
59	SHARON	COMPLETE	COMPLETE	
63	SHARON	COMPLETE	COMPLETE	
83	SHARON	COMPLETE	COMPLETE	
84	SHARON	COMPLETE	COMPLETE	
4	STANDARD	COMPLETE	COMPLETE	
51	STANDARD	COMPLETE	COMPLETE	
67	STANDARD	COMPLETE	COMPLETE	
73	STANDARD	COMPLETE	COMPLETE	
75	STANDARD	COMPLETE	COMPLETE	
78	STANDARD	COMPLETE	COMPLETE	
3	SHARON	PARTIAL	PARTIAL	
22	SHARON	PARTIAL	PARTIAL	
29	SHARON	PARTIAL	PARTIAL	
35	SHARON	PARTIAL	PARTIAL	
39	SHARON	PARTIAL	PARTIAL	
53	SHARON	PARTIAL	PARTIAL	
57	SHARON	PARTIAL	PARTIAL	
58	SHARON	PARTIAL	PARTIAL	
60	SHARON	PARTIAL	PARTIAL	
62	SHARON	PARTIAL	PARTIAL	
64	SHARON	PARTIAL	PARTIAL	
66	SHARON	PARTIAL	PARTIAL	
70	SHARON	PARTIAL	PARTIAL	
71	SHARON	PARTIAL	PARTIAL	
76	SHARON	PARTIAL	PARTIAL	
5	STANDARD	PARTIAL	PARTIAL	
15	STANDARD	PARTIAL	PARTIAL	
21	STANDARD	PARTIAL	PARTIAL	
34	STANDARD	PARTIAL	PARTIAL	
36	STANDARD	PARTIAL	PARTIAL	
44	STANDARD	PARTIAL	PARTIAL	

45	STANDARD	PARTIAL	PARTIAL	
49	STANDARD	PARTIAL	PARTIAL	
65	STANDARD	PARTIAL	PARTIAL	
81	STANDARD	PARTIAL	PARTIAL	
85	STANDARD	PARTIAL	PARTIAL	
86	STANDARD	PARTIAL	PARTIAL	
91	STANDARD	PARTIAL	PARTIAL	
56	SHARON	PROGRESSION	PROGRESSION	
82	SHARON	PROGRESSION	PROGRESSION	
11	STANDARD	PROGRESSION	PROGRESSION	
31	STANDARD	PROGRESSION	PROGRESSION	
46	STANDARD	PROGRESSION	PROGRESSION	
48	STANDARD	PROGRESSION	PROGRESSION	
50	STANDARD	PROGRESSION	PROGRESSION	
69	SHARON	**STABLE**	**PROGRESSION**	**X**
72	SHARON	**STABLE**	**PROGRESSION**	**X**
88	SHARON	**STABLE**	**PROGRESSION**	**X**
10	STANDARD	**STABLE**	**PROGRESSION**	**X**
6	STANDARD	**COMPLETE**	**STABLE**	**X**
41	STANDARD	**COMPLETE**	**STABLE**	**X**
8	SHARON	**PARTIAL**	**STABLE**	**X**
26	SHARON	**PARTIAL**	**STABLE**	**X**
40	SHARON	**PARTIAL**	**STABLE**	**X**
79	SHARON	**PARTIAL**	**STABLE**	**X**
7	STANDARD	**PARTIAL**	**STABLE**	**X**
9	STANDARD	**PARTIAL**	**STABLE**	**X**
14	STANDARD	**PARTIAL**	**STABLE**	**X**
33	STANDARD	**PARTIAL**	**STABLE**	**X**
43	STANDARD	PARTIAL	STABLE	
1	SHARON	STABLE	STABLE	
68	SHARON	STABLE	STABLE	
74	SHARON	STABLE	STABLE	
12	STANDARD	STABLE	STABLE	
27	STANDARD	STABLE	STABLE	
87	STANDARD	STABLE	STABLE	

Table S5. Pain response considering NRS modifications of 1 point

	COMPLETE RESPONSE	PARTIAL RESPONSE	STABLE DISEASE	PROGRESSION
STANDARD	8 (22,9%)	20 (57,1%)	2 (5,7%)	5 (14,3%)
SHARON	11 (28,9%)	22 (57,9%)	2 (5,3%)	3 (7,9%)

Figure S3. Patients lost to follow-up before, during and after COVD pandemic. The COVID period is defined from February 2020 to the end of December 2020, in accordance with the main pandemic peaks and lockdown restrictions in Italy. The Pre and Post-COVID periods precede and follow this time interval.

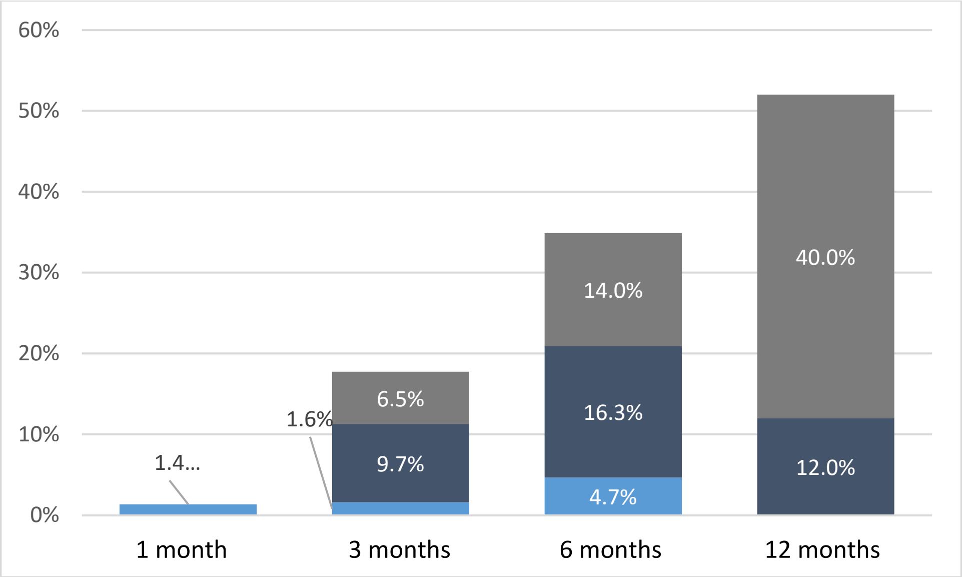

	1 month	3 months	6 months	12 months
PRE-COVID	1	1	2	0
COVID	0	6	7	3
POST-COVID	0	4	6	10

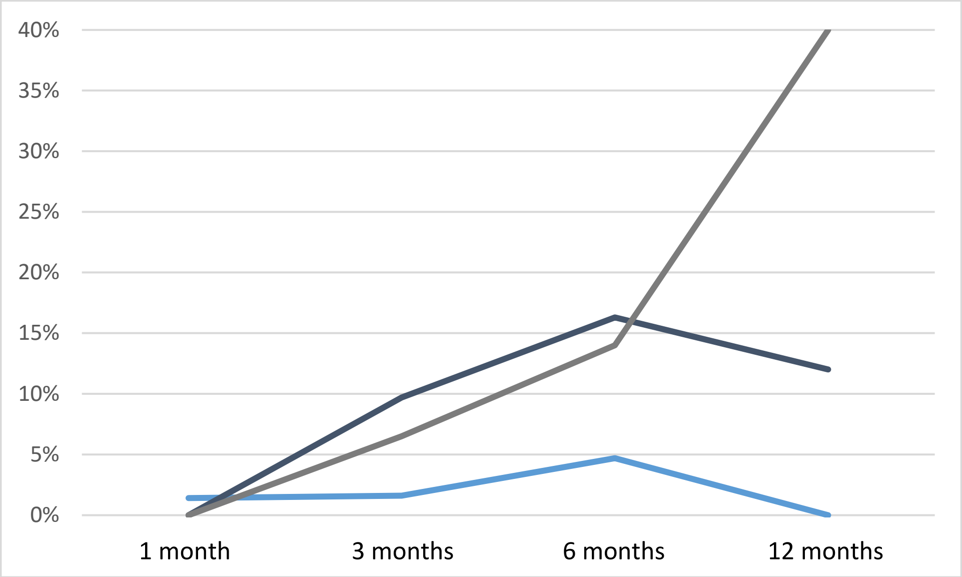

Legend: Pre-COVID | COVID | Post-COVID

The percentage are referred to patients still alive at each timepoint.

Table S6. Type and grade of toxicities

			Total toxicities registered, n: 38	Standard arm, n: 23	(% on pts, 41)	SHARON arm, n: 15	(% on pts, 42)
Type of toxicity	Upper GI	grade 1	9	6	14,6%	3	7,1%
		grade 2	3	3	7,3%	0	0,0%
	Lower GI	grade 1	8	2	4,9%	6	14,3%
		grade 2	1	1	2,4%	0	0,0%
	Pharynx/Esophagus	grade 1	5	4	9,8%	1	2,4%
	Skin	grade 1	2	0	0,0%	2	4,8%
	Genitourinary	grade 1	1	1	2,4%	0	0,0%
	Fatigue	grade 2	3	2	4,9%	1	2,4%
	Hematological	grade 1	1	1	2,4%	0	0,0%
		grade 2	1	1	2,4%	0	0,0%
		grade 3	1	0	0,0%	1	2,4%
	Spinal cord	grade 3	1	0	0,0%	1	2,4%
	Bone	grade 4	2	2	4,9%	0	0,0%

		Total population, n: 83	% on population*	Standard arm, n: 41	% on population standard arm*	SHARON arm, n: 42	% on population SHARON arm*
Toxicity per grade	Grade 0	51	61,4%	22	53,7%	29	69,0%
	Grade 1	26	31,3%	14	34,1%	12	28,6%
	Grade 2	8	9,6%	7	17,1%	1	2,4%
	Grade 3	2	2,4%	0	0,0%	2	4,8%
	Grade 4	2	2,4%	2	4,9%	0	0,0%

*sum of % higher than 100%, accounting for multiple toxicity per patients.
